# Multiomics Strategy Reveals the Mechanism of Action and Ameliorating Effect of Deer Velvet Antler Water Extracts on DSS-Induced Colitis

**DOI:** 10.3390/biomedicines11071913

**Published:** 2023-07-06

**Authors:** Ying-Kai Hung, Shang-Tse Ho, Ching-Yun Kuo, Ming-Ju Chen

**Affiliations:** 1 Department of Animal Science and Technology, National Taiwan University, Taipei 106, Taiwan; 2 Department of Wood Based Materials and Design, National Chiayi University, Chiayi 600, Taiwan; 3 Taiwan Livestock Research Institute, Council of Agriculture, Tainan 712, Taiwan

**Keywords:** velvet antler, inflammatory bowel disease, inflammatory response, intestinal barrier integrity, microbiota

## Abstract

Velvet antler is a precious traditional Chinese medicine used for thousands of years. This study investigated the anti-colitis effects of water extracts of Formosan sambar deer (SVAE) and red deer (RVAE) to identify the possible mechanisms and the bioactive compounds using a dextran sulfate sodium (DSS)-induced colitis mouse model. The mechanism of action and the ameliorating effects of SVAE and RVAE on DSS-induced colitis were evaluated using a mouse model. Ultra-high performance liquid chromatography-mass/mass and gas chromatography-mass/mass were applied to identify the bioactive components of the SVAE and RVAE water extracts. The results revealed that both high-dose SVAE and RVAE could ameliorate the symptoms of colitis due to reduced systemic inflammatory responses, enhanced intestinal barrier integrity by restoration of tight junction proteins, and improved gut dysbiosis. The potentially bioactive components of SVAE and RVAE were identified as small molecules (<3 kDa). Further identification by untargeted metabolomics analysis suggested that l-carnitine, hypoxanthine, adrenic acid, creatinine, gamma-aminobutyric-lysine, oleic acid, glycine, poly-γ-glutamic acid, and eicosapentaenoic acid in VAWEs might be involved in ameliorating the symptoms of colitis. This study provided evidence for the potential usage of SVAE and RVAE as anti-colitis agents.

## 1. Introduction

Inflammatory bowel disease (IBD) including ulcerative colitis and Crohn’s disease is characterized by relapsing disorders of abnormal inflammation of the gastrointestinal tract, a leaky gut, and dysbiosis of the microbiome [1,2]. In IBD patients, the risk of colorectal cancer and small bowel cancer increases two- to eight-fold and has been continuously increasing globally in the past decades [3,4]. Due to the complex interaction between the host immune response and the homeostasis of the gut microbiota, sophisticated reciprocal causation between impaired gut permeability, and dysbiosis caused by translocation of bacteria, the etiology of those disorders remains unclear [2,5,6].

There are two therapeutic approaches for IBD: (1) optimized anti-inflammatory treatment involving corticosteroids and monoclonal antibodies which target aberrant immune progression, and (2) microbiome-modulating interventions, such as antibiotics and probiotic supplements [7]. Nevertheless, these treatments are not always as good as expected [2,8,9]. In addition, exposure to antibiotics may suppress the growth of *Bacteroides*, *Lachnospiraceae*, and *Ruminococcaceae*, thereby deteriorating colitis and causing relapse with multiple side effects [10,11,12]. Although fecal microbiota transplantation (FMT) has therapeutic effects on colitis caused by *C. difficile* infection, the associated risks have resulted in the United States Food and Drug Administration (FDA) issuing a warning advising against its use [13]. In other words, the limitations of the above therapies have not yet been resolved, so alternatives are needed.

Velvet antler (VA) is a cartilaginous tissue without full calcification that has been used in traditional Chinese medicine for thousands of years. Based on the traditional theory of Chinese medicine, VA can strengthen the functions of the liver and kidneys [14]. Several pharmacological properties of VA extracts have been demonstrated, such as anti-osteoporosis, anti-inflammation, and wound healing-promoting effects [14,15]. Specifically, in collagen-induced arthritis, the expression of pro-inflammatory cytokines cyclooxygenase-2 (COX-2), tumor necrosis factor-α (TNF-α), and interferon-γ (IFN-γ) were reduced in arthritic joints on treatment with VA water extracts [16]. In addition, interleukin-6 (IL-6) and IL-17F were downregulated after treatment with VA extracts in an allergic asthma model [17]. VA extracts can induce the differentiation of neural stem cells [18], and ameliorate injury in cardiac microvascular endothelial cells [19] but few studies have ever investigated the anti-colitis effect of VA.

Dietary supplements utilizing VAs commonly originate from Formosan sambar deer and red deer species. In our previous studies, the VA water extracts from Formosan sambar deer reduce the production of TNF-α and IL-6, thereby relieving inflammation in a *Staphylococcus aureus*-infected model [20]. In our previous study, both velvet antler water extracts (VAWE) could also augment barrier integrity in colonic epithelial cells (Caco-2) through upregulation of occludin and claudin1 expression, and production of C-C chemokine ligand 20 (CCL-20) [21], suggesting a possible intestinal protecting effect of VA to restore digestive tract function. Thus, the present study investigated the anti-colitis effects of the Formosan sambar deer water extracts (SVAE) and red deer water extracts (RVAE) by evaluating the anti-inflammatory effect and mucosal integrity in a mouse model. To clarify the possible mechanism and bioactivated components involved in VA, the colonic microbiome and the VA metabolome were analyzed. The biomarker interactions between the microbiome and epithelial integrity were also investigated.

## 2. Results

### 2.1. VAWEs Ameliorated the Symptoms in DSS-Induced Colitis

**Figure 1:**
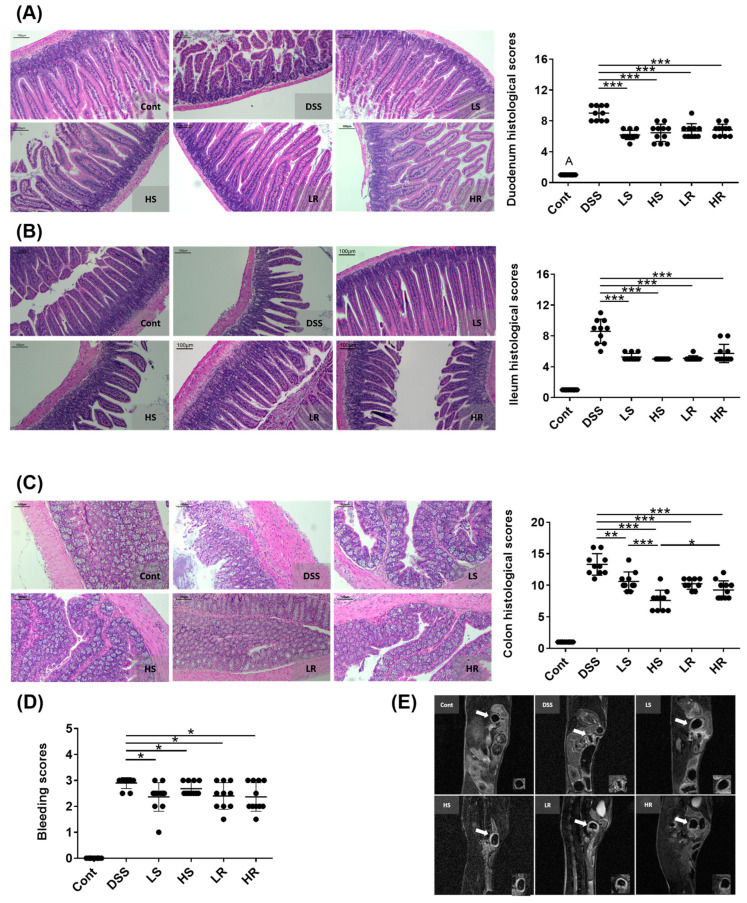
Effects of VAWEs on the symptoms of colitis in a DSS-induced colitis mouse model. Images of H&E staining for (**A**) duodenum, (**B**) ileum, and (**C**) colon sections were quantitatively analyzed with an individual pathological injury score. Scale bar = 100 μm. (**D**) Scores of occult bleeding evaluation in feces; and (**E**) MRI imaging of proximal colon wall thickness. The data are presented as mean ± SD (n = 8–10). Symbols indicate significant difference between the VAWE-treated group when compared with the DSS-treated group (* *p* < 0.05, ** *p* < 0.01, *** *p* < 0.001). Groups: control (Cont), DSS control (DSS), low dosage of SVAE (LS), high dosage of SVAE (HS), low dosage of RVAE (LR), and high dosage of RVAE (HR).

Firstly, we investigated the protective effect of SVAE and RVAE on colitis in vivo. After 7 days of DSS administration, although weight loss and decreased food intake were not resolved with the VAWEs treatment (Figure S1A,B), there was significantly lower inflammatory infiltration, crypt damage, goblet cell depletion, stool bleeding score, and structural destruction in histological analyses of the duodenum, ileum, and colon sections compared to the DSS group (*p* < 0.05) (Figure 1A–D). On day 19, a thinner colon wall was observed in all VAWE-treated groups compared to the DSS group, indicating reduced inflammation (Figure 1E).

### 2.2. VAWEs Reduced the Systemic Inflammatory Response in DSS-Induced Injury

**Figure 2:**
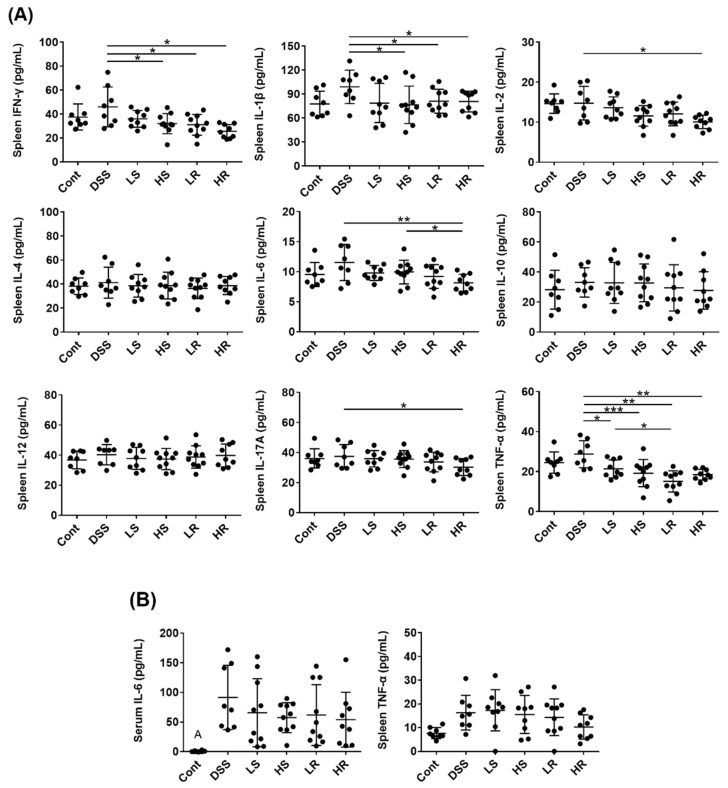
Effects of VAWEs on the inflammatory biomarkers in a DSS-induced colitis mouse model. Cytokine analysis in the (**A**) spleen and (**B**) serum. The data are presented as mean ± SD (n = 8–10). * *p* < 0.05, ** *p* < 0.01, *** *p* < 0.001 between the VAWE-treated group compared with the DSS-treated group. Groups: control (Cont), DSS control (DSS), low dosage of SVAE (LS), high dosage of SVAE (HS), low dosage of RVAE (LR), and high dosage of RVAE (HR).

We then clarified the possible mechanisms involved in the preventive effect of VAWEs on colitis. Systematic inflammation was examined by the detection of serum and spleen cytokines (Figure 2). In the spleen samples, HS, LR, and HR groups showed significantly lower levels of TNF-α, IL-1β, and IFN-γ than the DSS group (*p* < 0.05) but only high-dose RVAE could reduce the production of IL-6, IL-2, and IL-17A (*p* < 0.05). However, there was no significant difference between VAWEs-treated groups and the DSS group in IL-4, IL-10, and IL-12 levels (Figure 2A). Serum IL-6 levels were reduced compared to the DSS-treated group but not significantly (Figure 2B).

### 2.3. VAWEs Restored the Tight Junction Associated Proteins against DSS Challenge

**Figure 3:**
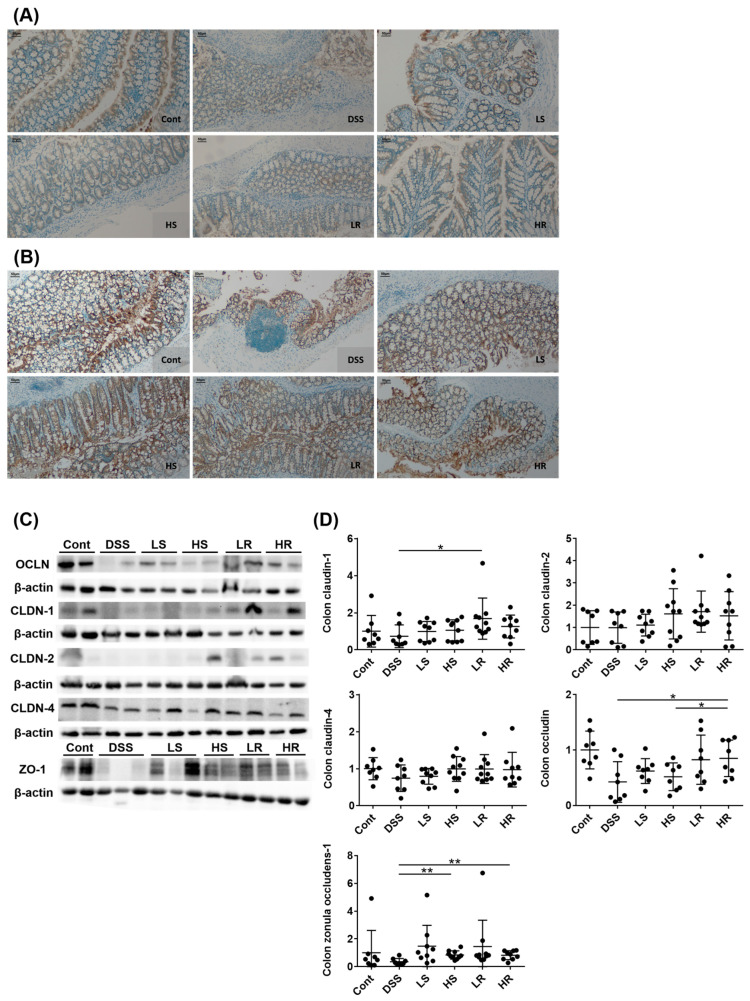
Effects of VAWEs on colonic tight junction proteins. (**A**,**B**) Representative immunohistochemical images of colonic occludin and claudin-1 staining, respectively. Scale bar = 50 μm. (**C**) Western blot analysis of occludin, claudin-1, claudin-2, claudin-4, and ZO-1. (**D**) Relative protein expression of occludin, claudin-1, claudin-2, claudin-4, and ZO-1 normalized to β-actin expression. The data are presented as mean ± SD (n = 8–10). * *p* < 0.05, ** *p* < 0.01 between the VAWE-treated group compared to the DSS-treated group. Groups: control (Cont), DSS control (DSS), low dosage of SVAE (LS), high dosage of SVAE (HS), low dosage of RVAE (LR), and high dosage of RVAE (HR).

In addition to inflammation, barrier integrity is another crucial biomarker of colitis and was evaluated by the immunoblot analysis of tight junction proteins in colon tissue. In the IHC staining images of colonic occludin and claudin-1 (brown dots), these proteins were tightly arrayed between epithelial cells in the Cont group, maintaining barrier integrity, whereas injured cells and severe lymphocyte infiltration were observed in the DSS group with loosely connected occludin and claudin-1. High-dose VAWEs prevented damage to tight junction proteins (Figure 3A,B). Western blotting validated the IHC findings (Figure 3C,D). RVAEs significantly upregulated the expression of colonic tight junction proteins, occludin (HR), claudin-1(LR), and ZO-1 (HR) (*p* < 0.05) compared to the DSS counterpart, with claudin-2 and claudin-4 showing a tendency to increase. For the SVAE intervention, only a significantly higher expression of colonic ZO-1 (*p* < 0.01) was observed in the HS group compared to the DSS group.

### 2.4. RVAE Maintained the Secretion of Short Chain Fatty Acids via the HIF-1α Pathway

**Figure 4:**
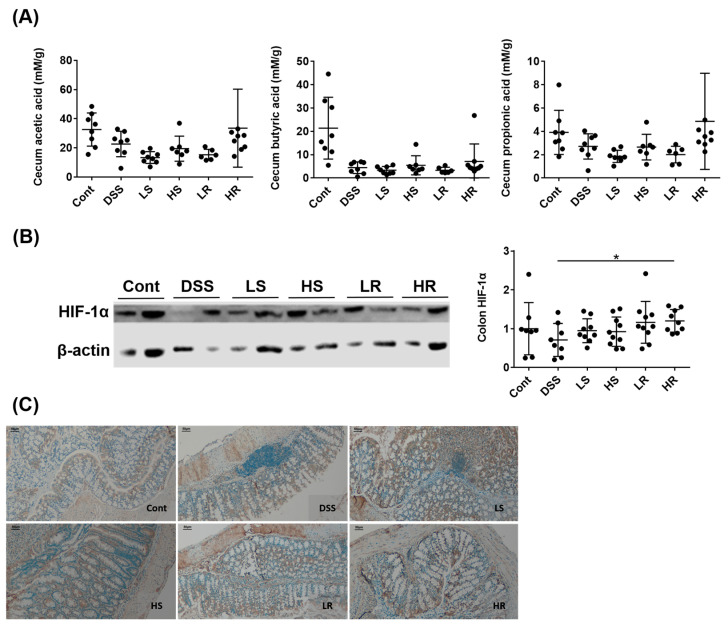
Effects of VAWEs on the biological pathways of junction proteins and the effect of the microbiome. (**A**) Short-chain fatty acid content in the cecum. (**B**) Western blot analysis of colonic HIF-1α. (**C**) Representative immunohistochemical images of colonic HIF-1α staining, scale bar = 50 μm. Protein was normalized to the β-actin expression and the data are presented as mean ± SD (n = 8–10). * *p* < 0.05 between the VAWE-treated group compared to the DSS-treated group. Groups: control (Cont), DSS control (DSS), low dosage of SVAE (LS), high dosage of SVAE (HS), low dosage of RVAE (LR), and high dosage of RVAE (HR).

The downstream metabolites of gut microorganisms and SCFA in the cecum were analyzed, showing that among all VAWEs-treated groups, only the HR group showed a trend for increased acetic acid and propionic acid levels (Figure 4A). Additionally, a biomarker for the depleting state of physiological hypoxia caused by the interaction between the microbiome effect and epithelial integrity called HIF-1α was analyzed by immunoblotting, showing that HIF-1α expression was significantly higher in the HR group than the colitis DSS group (*p* < 0.01) in line with the results regarding microbial-derived SCFA (Figure 4B,C).

### 2.5. RVAE Partially Restored the Homeostasis of the Gut Microbiota in DSS-Induced Colitis via Alteration of Enriched Taxa

The microbiome results in cecum content showed that intervention with VAWEs did not affect the alpha diversity parameters chao1 and Shannon (Figure S2A). Beta diversity, evaluated by PLS-DA analysis, indicated that all groups induced by DSS were separated from the normal control group, suggesting the severe alteration of the intestinal flora induced by DSS. Thus, we excluded the effect dominated by the Control group and performed the same PLS-DA analysis, showing that the VAWEs-treated groups could be separated from the DSS group. Furthermore, HS and HR overlapped in the PLS-DA analysis (Figure S2B).

**Figure 5:**
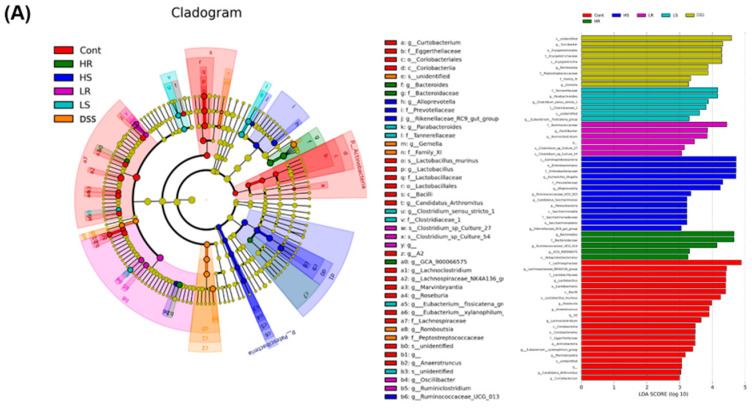

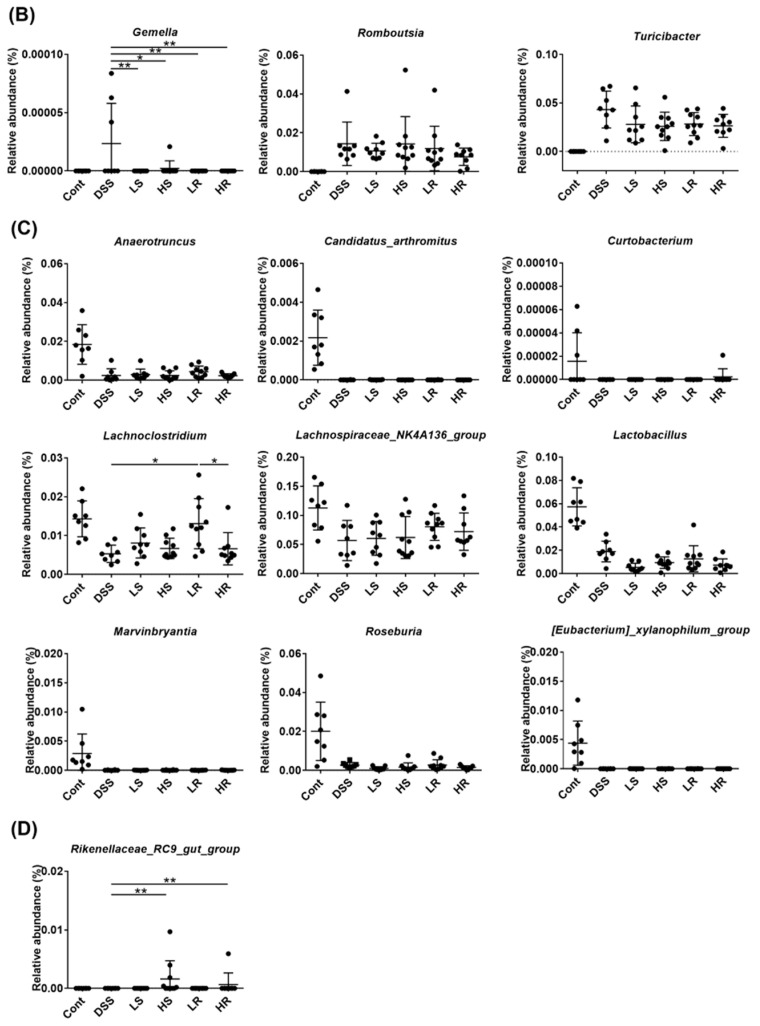
Effects of different VAWEs on the microbiome in the DSS-induced colitis mouse model. (**A**) Linear discriminant analysis effect size analysis (LEfSe) and histogram of the 3.0 LDA scores of gut microbiota. The relative abundance of selected microbial biomarkers in the (**B**) DSS group, (**C**) Control group, and (**D**) HS group. The values are mean ± SD (n = 8–10). Significant differences were observed when comparing the VAWE-treated group with the DSS-treated group * *p* < 0.05, ** *p* < 0.01. Groups: control (Cont), DSS control (DSS), low dosage of SVAE (LS), high dosage of SVAE (HS), low dosage of RVAE (LR), and high dosage of RVAE (HR).

Since the NGS results indicated that the intervention of various VAWEs could be distinguished at the gastrointestinal bacteria level, bacterial biomarkers were identified by the linear discriminant analysis (LDA) effect size (LEfSe) algorithm. Under the 3.0 LDA score, a total of 60 influential taxonomic clades were recognized, including 28 bacterial genera, and six bacterial species (Figure 5A). Several biomarkers identified in colitis mice (DSS group) were three genera (*Turicibacter*, *Romboutsia*, and *Gemella*), while nine genera (*Anaerotruncus*, *Candidatus*, *Curtobacterium*, *Lachnoclostridium*, *Lachnospiraceae_NK4A136_group*, *Lactobacillus*, *Marvinbryantia*, *Roseburia*, *[Eubacterium]_xylanophilum_group*), and one species (*Lactobacillus_murinus*) were the most influential taxa in the control group. For VAWE-treated groups, the biomarkers identified in taxa genera and species for each group were: three genera (*Parabacteroides*, *Clostridium_sensu_stricto_1*, and *Eubacterium_fissicatena_group*) in the LS group, five genera (*Escherichia_Shigella*, *Alloprevotella*, *Ruminococcaceae_UCG_013*, *Candidatus_Saccharimonas*, and *Rikenellaceae_RC9_gut_group*) in the HS group, five genera (*Oscillibacter*, *Ruminococcaceae*, *Ruminiclostridium*, *Clostridium_sp_culture_27*, and *Clostridium_sp_culture_54*) in the LR group, and two genera (*Bacteroides* and *Ruminococcaceae_UCG_014*) in the HR group.

The relative abundances of the bacterial biomarkers associated with the DSS group were consistent with the above findings (Figure 5B). The relative abundances of all three bacterial biomarkers (genus levels) identified in the DSS group were significantly higher than that of the control group (*p* < 0.05). VAWEs intervention could significantly downregulate *Gemella* (*p* < 0.05) and a tendency was shown in the sense of reducing the two other genera. Conversely, a significantly higher relative abundance of all ten bacterial biomarkers identified in the normal control group was observed when compared with the DSS group (*p* < 0.05). Intervention with low-dose and high-dose RVAEs could significantly upregulate the relative abundance of *Lachnoclostridium* and *Curtobacterium*, respectively (*p* < 0.05) (Figure 5C). Additionally, both high-dose RVAE and SVAE caused significantly higher *Rikenellaceae_RC9_gut_group* than that in the other groups (*p* < 0.05) (Figure 5D).

**Figure 6:**
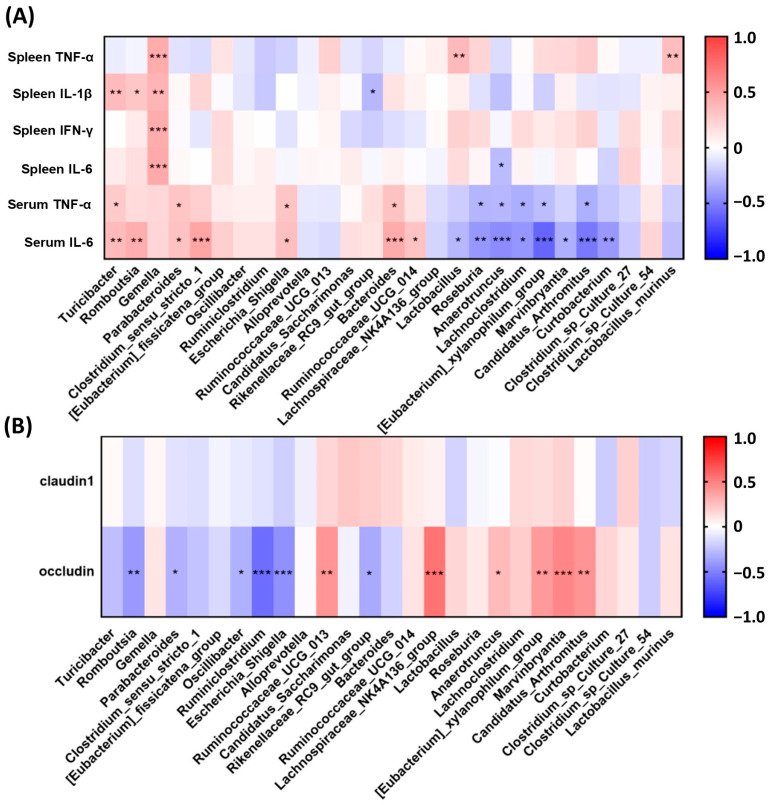
Spearman’s correlation analysis of predominant genus, species level of identified bacteria and the inflammatory cytokines (**A**), and tight junction protein (**B**), respectively. Columns with symbols indicate significant differences in correlation coefficient (r) * *p* < 0.05, ** *p* < 0.01, *** *p* < 0.001.

After identifying the bacterial biomarkers, we illustrated the correlation of inflammation and mucus integrity proteins with the bacterial biomarkers at the genus and species levels (Figure 6). The genera enriched in the colitis DSS group such as Turicibacter, Romboutsia, and Gemella were positively and negatively correlated with the inflammatory cytokines and junction proteins, respectively. Conversely, the genera enriched in the control group, including Lachnospiraceae_NK4A136_group, Lactobacillus, Roseburia, Anaerotruncus, Lachnoclostridium, Eubacterium_xylanophilum_group, Marvinbryantia, Candidatus_Arthromitus, and Curtobacterium, demonstrated negative correlations with serum TNF-α and IL-6, and positive correlation with occludin. The three (Parabacteroides, Clostridium_sensu_stricto_1, and Escherichia_Shigella) and two (Parabacteroides and Escherichia_Shigella) biomarkers associated with SVAE intervention groups were positively and negatively correlated with serum inflammatory cytokines and occludin, respectively. Conversely, biomarker Rikenellaceae_RC9_gut_group in the HSs group was negatively correlated with serum TNF-α and IL-6, and Ruminococcaceae_UCG_013 was positively correlated with occludin. For biomarkers in the RVAE intervention groups, Bacteroides and Ruminococcaceae_UCG_014 demonstrated a positive correlation with serum inflammatory cytokines.

### 2.6. The Molecular Weight of Active Components in SVAE and RVAE Was Less than 3 kDa

**Figure 7:**
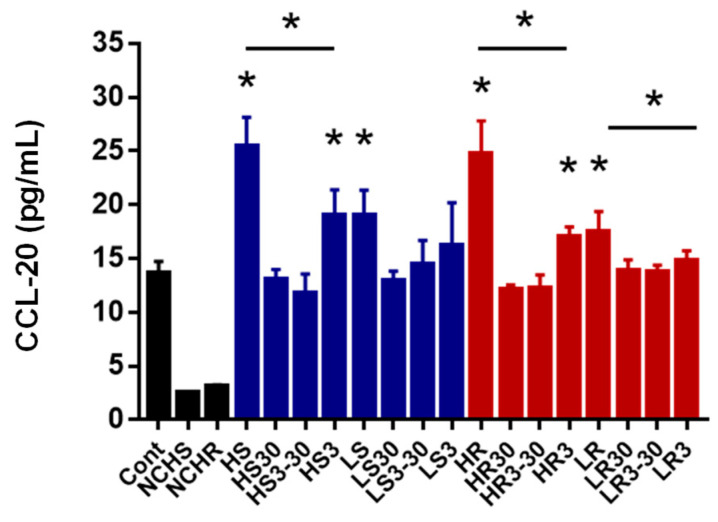
Effects of different molecular fragments of VAWEs on CCL-20 production in Caco-2 cells. The values are mean ± SD (n = 3), (* *p* < 0.05). Cont: control group, NCHS: negative control of high dosage of SVAE, NCHR: negative control of high dosage of RVAE, HS: high dosage of SVAE, HS30: Molecules with a molecular weight greater than 30 kDa in high dosage SAVE, HS3~30: Molecules with a molecular weight between 3 and 30 kDa in high dosage SAVE, HS3: Molecules with a molecular weight below 3 kDa in high dosage SAVE, LS: low dosage of SVAE, LS30: Molecules with a molecular weight greater than 30 kDa in low dosage SAVE, LS3~30: Molecules with a molecular weight between 3 and 30 kDa in low dosage SAVE, LS3: Molecules with a molecular weight below 3 kDa in low dosage SAVE, HR: high dosage of RVAE, HR30: Molecules with a molecular weight greater than 30 kDa in high dosage RAVE, HR3~30: Molecules with a molecular weight between 3 and 30 kDa in high dosage RAVE, HR3: Molecules with a molecular weight below 3 kDa in high dosage RAVE, LR: low dosage of RVAE, LR30: Molecules with a molecular weight greater than 30 kDa in low dosage RAVE, LR3~30: Molecules with a molecular weight between 3 and 30 kDa in low dosage RAVE, LR3: Molecules with a molecular weight below 3 kDa in low dosage RAVE.

We then evaluated the active components of VAWEs in vitro by the CCL-20 level, which is a crucial marker involved in the restitution of colonic epithelial cells. As shown in Figure 7, the production of CCL-20 was mainly induced by fragments under 3 kDa (HS3 and HR3) in weight. However, compared to the crude extracts of SVAE (HS group) and RVAE (HR and LR groups), CCL-20 production by the HS3 and HR3 groups was significantly lower.

### 2.7. Potential Bioactive Components of SVAE and RVAE Were Identified by Untargeted UHPLC-MS/MS and GC-MS/MS

UHPLC-MS/MS and GC-MS/MS were performed to identify the small components of SVAE and RVAE, identifying 4250 and 98 molecules, respectively. When comparing RVAE and SVAE, 99.88% of the components were identical, with 2656 molecules (61.09%) in RVAE having higher relative abundance than in SVAE. Only five unique components were found in SVAE, including campesterol 6′-hexadecanoylglucoside, and CE(20:5(5Z,8Z,11Z,14Z,17Z). Moreover, 836 molecules were identified without isomers with higher accuracy, and the 10-fold greater or lower relative abundance in RVAE than in SVAE was composed of 129 and 45 molecules, respectively, accounting for only 4% of the identified molecules.

The top 20 dominant molecules identified in SVAE and RVAE covered 63.14% and 64.04% of the total components in the positive ion UHPLC-MS/MS (Table 1), 59.10% and 62.27% in the negative ion UHPLC-MS/MS (Table 2), and 89.15% and 90.36% of GC-MS/MS (Table 3), respectively. Among them, palmitic acid, l-carnitine, and oleic acid were ranked the top three in terms of their relative abundance accounting for 25.66%, 19.32%, and 11.83% in SVAE, and 24.12%, 18.49%, and 14.57% in RVAE, respectively. Furthermore, the ratio of individual components in RVAE and SVAE is presented in Tables S1 and S2 according to the ranking from the computational quotient.

## 3. Discussion

The present study demonstrated the anti-colitis effect of VA extracts from red deer and Formosan sambar deer by integrating the tripartite mechanisms of the aberrant inflammatory response, epithelial mucus integrity, and microbiome effect. The colitis symptoms induced by DSS including mucosal neutrophil infiltration and damage to the colon were significantly attenuated in all VA-treated groups. The abnormal enhancement of pro-inflammatory cytokines is an indicator of colitis [2]. The decreased spleen TNF-α, IL-1β, IFN-γ, and IL-6 levels in the VA-treated mice reduced the inflammatory cascade and was in line with our previous finding in RAW 264.7 cells [22]. Earlier reports also revealed that blocking TNF-α, IL-1β, or IL-6 caused an anti-colitis effect [2,23,24,25,26]. Additionally, both IL-2, the upstream initiator of Th1 response [27], and IL-17, induced by IL-1β [24], were significantly reduced only in HR groups. Nevertheless, no IL-10 activation was observed, which functions as an anti-inflammatory mediator to downregulate the expression of Th1-derived cytokines [28,29], and promote IL-4, classified as Th2-derived cytokines to reduce colitis [30,31].

Tight junction proteins contribute to the physical barrier and were suppressed by increased TNF-α and IFN-γ production synergistically [32,33,34,35]. Restoration of junctional proteins in colon mucus by pre-treatment with VA could strengthen barrier function and maintain gut permeability to reduce the damage caused by DSS, which is consistent with our in vitro data [21]. Although none of the therapies targeting junction proteins have currently been approved [36], this finding suggests that the VA water extraction might provide another potential treatment for intestinal colitis through the upregulation of tight junction proteins to strengthen the gut barrier.

The upregulation of SCFA and the expression of colonic HIF were also involved in the intestinal barrier-protecting effect via augmenting tight junction proteins. SCFAs enhance barrier integrity via the claudin-1 pathway [37,38], elevate IL-10 production through activating Treg differentiation [39], and improve the efficacy of broad antibiotic therapy in colitis [40]. Meanwhile, in the pathogenesis of colitis with depletion of epithelial O_2_, changes in colonic HIF-1α expression are related to barrier permeability through the claudin-1 pathway [41,42,43]. Furthermore, the alteration of intestinal microbiota is not only reflected in the SCFA levels, as the expression of colonic HIF-1α was also affected by fluctuations in oxygen concentration in the lower gastrointestinal tract, resulting in microbial fermentation and digestion in mucosal surfaces [44,45]. The significantly higher colonic SCFA and HIF-1α expression in the HR group indicated that a high dose of red deer VA extract could augment the tight junction protein expression through modulation of important gut metabolites and regulation of HIF-1α protein expression. This also suggests that the microbial composition might be directly affected by VAWE supplementation.

NGS analysis revealed the role of gut microbiota on the anti-colitis effect of VAWEs, as the VAWE intervention restored the dysbiosis caused by DSS. The community structure of microbiota shifted to re-establish homeostasis, as shown in the PLS-DA analysis. The results of LEfSe and Spearman’s correlation indicated that the shift in gut microbiota might be motivated by the following genera: Gemella, Ruminococcaceae_UCG_014, Clostridium_sensu_stricto_1, Curtobacterium, Rikenellaceae_RC9_gut_group, and Lachnoclostridium, which were significantly correlated with pro-inflammatory cytokines and junction proteins. Except for Curtobacterium, all other genera have been reported to be involved in intestinal disease or protection.

Gemella is found in patients with Crohn’s disease [46,47] and was increased in the DSS group and significantly decreased after VAWE intervention. Ruminococcaceae_UCG_014 was significantly decreased in the LR groups, which was consistent with previous studies of 5-aminosalicylic acid treatment for IBD patients [48,49]. The alteration of Clostridium_sensu_stricto_1 and Lachnoclostridium in the LR group was paralleled with the observation in colitis patients receiving FMT therapy [50,51]. Although the role of Rikenellaceae_RC9_gut_group on colitis was not unanimously described [52,53], certain families of Rikenellaceae and Lachnoclostridium could synthesize butyrate [54,55]. Curtobacterium strains, which have been isolated from human clinical specimens [56], were first shown to experience a positive effect on anti-colitis after intervention with high-dose RVAE. However, the physical role of this genus on intestinal protection still needs to be investigated. Additionally, Escherichai_shigella, the representative bacteria attacking intestinal epithelial cells in colitis [57] also decreased in the HR group.

We then identified the active pharmacological components of VAWEs. First, we revealed that the active fractions composed of small molecules (<3 kDa) in both SVAE and RVAE significantly upregulated CCL-20 production. This chemokine is associated with actin cytoskeleton reorganization and contributes to the mucosal healing process and epithelial cell migration [58]. Several small bioactive components (about 3 kDa) from VA extracts have been reported to elevate Th1/Th2 cytokine production [59], stimulate wound healing in vitro [60], and enhance osteoblast proliferation [61].

UHPLC-MS/MS and GC-MS/MS analysis revealed similar components in different proportions in the RVAE and SAVE. Among the top 20 identical molecules identified in both RVAE and SAVE, l-carnitine with its metabolic forms (acetylcarnitine, and 2-methylbutyroylcarnitine) [62], hypoxanthine [63], adrenic acid [64], and stearic acid [65,66] demonstrated a protective effect on intestinal mucosa via anti-inflammation. Creatinine [67,68], gamma-aminobutyryl-lysine [69], oleic acid [70,71], glycine [72,73], and poly-γ-glutamic acid [74] have been reported to attenuate colitis through an anti-inflammatory effect, upregulating intestinal mucosa protein expression, and/or promoting SCFA production. The identical dominant molecules identified in both RVAE and SAVE could be important bioactive components responsible for the anti-colitis effect.

However, the anti-colitis effect of SVAE and RVAE varied, possibly due to the different relative abundance of identical molecules and unique components in RVAE. Among the identical molecules with a ratio greater than 50-fold, eicosapentaenoic acid is one of the crucial n-3 polyunsaturated fatty acids that improves the clinical symptoms of ulcerative colitis, including reduction in fecal calprotectin level, alleviation of mucosal inflammation, and promotion of goblet cell differentiation [75,76], which might contribute to the anti-colitis effect of RVAE. The anti-colitis roles of two unique molecules, campesterol 6′-hexadecanoyl glucoside, and CE (20:5 (5Z, 8Z, 11Z, 14Z, 17Z), in RVAE are not clear.

## 4. Materials and Methods

### 4.1. Reagents

Dextran sulfate sodium (DSS) was purchased from MP BioChemicals (molecular weight: 36,000-50,000 Da; Santa Ana, CA, USA). DMEM (Dulbecco’s Modified Eagle Medium) medium, fetal bovine serum (FBS), and other cell culture reagents were obtained from Corning (Tewksbury, MA, USA). All chemicals and solvents used in this study were analytical grade.

### 4.2. Preparation of Velvet Antler Water Extracts

Formosan sambar deer were reared within 65–70 days and VA samples were kindly provided from Kaohsiung Animal Propagation Station, Taiwan Live Stock Research Institute (Pintong, Taiwan). Red deer were reared for 70–75 days and VA samples were purchased from Feng Ying Deer Ranch (Tainan, Taiwan). The VAWEs were prepared as described previously [19]. In brief, fresh VA samples were sliced, stored at −80 °C, then dehydrated using a freeze dryer (Kingmech Co. Ltd., Taipei, Taiwan). VA powder was extracted by immersing in an ultrasonic cleaner (Delta, Co. Ltd., Taipei, Taiwan) with water (50 g/L) at 4 °C and 15 min rest from cooling every hour. The supernatants were further dehydrated by lyophilization to obtain SVAE and RVAE.

### 4.3. DSS-Induced Colitis Animal Model

Female C57BL/6 mice (9 weeks old) were purchased from BioLasco Taiwan Co. Ltd. (Ilan, Taiwan) and housed in a specific pathogen-free environment, two mice per cage, under controlled air conditions and a 12-h light–dark cycle. All animal experiments were performed per the relevant guidelines and legal requirements (certification number: 201800166) and approved by the Institutional Animal Care and Use Committee (IACUC) (National Taiwan University, Taipei, Taiwan). After one week of acclimatization, the mice were randomly divided into six groups: control (Cont), DSS control (DSS), low dosage of SVAE (LS), high dosage of SVAE (HS), low dosage of RVAE (LR), and high dosage of RVAE (HR). Cont and DSS groups were orally gavaged with 200 μL of phosphate-buffered saline (PBS 1X, Hyclone*^®^*, Logan, UT, USA). LS and HS groups were orally gavaged with 200 μL of 100 mg/kg and 200 mg/kg SVAE, respectively, while LR and HR groups were orally gavaged with 200 μL of 100 mg/kg and 200 mg/kg RVAE, respectively. After 14-day pretreatment, the drinking water of the DSS, LS, HS, LR, and HR groups was replaced with the 2.5% DSS solution for 7 days to induce colitis. During these seven days, SAVE and RAVE were continuously orally gavaged. The body weight and feed intake were recorded during the experimental period. On day 19, 3 mice from each group were randomly selected for colon wall thickness measurement by Magnetic Resonance Imaging (MRI). At the end of the study, the mice were anesthetized with isoflurane, then sacrificed to harvest the blood, feces, spleen, duodenum, ileum, colon, and cecum contents and tissue for further analysis. The DSS-induced colitis model was conducted with slight modifications from the previous study [22,77,78].

### 4.4. Magnetic Resonance Imaging (MRI) Colon Monitoring

MRI was performed using the protocol described by Beltzer et al. [79]. Briefly, selected mice were anesthetized with 1.5% isoflurane, and a soft hollow tube was inserted into the rectum. The mice were placed on the 7T small scanner. First, the longitudinal section photography was taken to find the enlarged shape of the cecum and then, the image of the digestive tract was traced to localize the colon. Detailed imaging of this area was performed using the following parameters: repetition time of 3000 ms, echo time 36 ms, slice thickness 1 mm, a field of view 2.56 × 2.56 cm^2^, matrix size 256 × 256, and total scan time of 384 s.

### 4.5. Fecal Bleeding Test

At the end of the study, the collected stools were scored using a fecal occult blood test (Beckman Coulter, Inc., Fullerton, CA, USA) according to the process described by Wirtz et al. [80].

### 4.6. H&E Tissue Staining and Immunohistological Staining

Fresh duodenum, ileum, and colon tissues were washed with PBS to remove cell debris and then fixed in 10% formaldehyde (J.T. Baker*^®^*, Center Valley, PA, USA). After overnight soaking, the tissues were embedded and prepared for H&E staining performed by Raya Biotech Ltd. (Taipei, Taiwan). The H&E stained slices were imaged under the microscope with the acquisition of three visual fields (AxioObser Z1, Gottingen, Germany). The pathological scores of each image were evaluated by a veterinarian according to the standard [81]. As for the immunohistological staining, fresh slices of colon tissue were heated to remove the wax at 62 °C and then rehydrated, blocked, and incubated with primary antibody, followed by secondary antibody by Raya Biotech Ltd. (Taipei, Taiwan).

### 4.7. Cytokine Detection in Serum and Spleen Tissue

The spleen tissue was lysed in 100 μL of radioimmunoprecipitation assay (RIPA) buffer containing 1% protease inhibitor, and 1% 0.5 M EDTA (Sigma-Aldrich, St. Louis, MO, USA). The supernatant was collected after centrifugation at 12,000× *g* for 30 min. The protein concentrations were quantified with the bicinchoninic acid (BCA) protein assay kit (Thermo Fisher Scientific, Waltham, MA, USA) before TNF-α, IFN-γ, IL-1β, IL-2, IL-4, IL-6, IL-10, IL-12, and IL-17A were detected by ELISA kits (R&D systems Inc., Mckinley, MN, USA) using 50 μg (in 100 μL RIPA buffer) of protein. The serum samples (100 μL) were measured using the same procedure without protein quantification.

### 4.8. Western Blot Analysis

The colon tissue was lysed by the same procedure as described for the ELISA assay of spleen tissues. After quantification, 20 μg of protein samples were loaded onto 8%, 10%, and 12% SDS-PAGE gels for analysis of the expression of occludin (ab216327), claudin1 (ab15098), claudin2 (ab53032), claudin4 (ab15104), Zonula occludens-1 (ZO-1) (ab96587), CCL-20 (ab9829), HIF-1α (ab2185), and β-actin (ab16039) as loading control (Abcam, Cambridge, MA, USA). After transferring onto PVDF membranes (Merck Millipore Ltd., Burlington, MA, USA), and soaking in the blocking buffer (Thermo Fisher Scientific, Waltham, MA, USA) for 15 min, the membranes were incubated with primary antibodies at 4 °C overnight. Then, the membrane was washed thrice with TBS-Tween buffer, and incubated in horseradish peroxidase (HRP)-labeled rabbit secondary antibodies (Abcam, Cambridge, MA, USA) for an hour. Finally, the membranes were immersed in western lighting ECL pro reagent (Perkin-Elmer, Waltham, MA, USA), and analyzed using the ChemiDoc Touch Imaging System (Bio-Rad, Hercules, CA, USA). The quantification of protein expression was performed in ImageJ (n = 8–10) [82].

### 4.9. Short-Chain Fatty Acid Analysis

The measurement of SCFAs in the cecum contents was modified from the previous study [83]. Briefly, with a procedure of multiple chemical reactions of derivatization, the samples were extracted in 1 mL of methanol and analyzed by HPLC (Jasco International Co. Ltd., Tokyo, Japan) with a C18 column (ReproSil 100 C18 5 μm, 250 × 4.6 mm, Dr. Maisch GmbH, Ammerbuch, Germany). The mobile phase was composed of acetonitrile, methanol, and ultrapure water [30:16:54, pH = 4.5, adjusted with 0.1% TFA (Sigma-Aldrich, St. Louis, MO, USA)]. The injection volume was 30 μL, the flow rate was 1.1 mL/min, the column temperature was 50 °C, and the detection wavelength was 400 nm.

### 4.10. DNA Extraction and Next-Generation Sequencing of Gut Microbiota

The bacterial genomic DNA in the cecum contents was extracted using the QIAamp DNA Stool kit (Qiagen, Hilden, Germany) following the manufacturer’s instructions. After quantification of the DNA samples using a Nanodrop 2000 spectrophotometer (Thermo Scientific, Waltham, MA, USA), the V3-V4 region of 16S ribosomal RNA was amplified with primers 341F (5′-CCTACGGGAGGCAGCAG-3′) and 806R (5′-GGACTACCAGGGTATCTAAT-3′) containing barcodes and sequenced on the Illumina MiSeq platform (San Diego, CA, USA). A representative sequence of each operational taxonomic unit (OTU) of taxonomic annotation was read and compared with the Ribosomal Database Project classifier v2.2. The indices of alpha diversity (Chao1 and Shannon), and beta diversity (partial least squares discrimination analysis, PLS-DA) were analyzed by QIIME v1.7.0 with R v2.15.3 software. Based on the score of linear discriminant analysis (LDA), effect size analysis (LEfSe) was applied for biomarker identification at the individual taxonomic level based on the enrichment abundance among groups.

### 4.11. Caco-2 Cell Culture

The human colonic epithelial cell line Caco2-C2BBe1 was purchased from Bioresource Collection and Research Center (BCRC, Hsinchu, Taiwan), and cultured in DMEM medium containing 10% heat-inactivated FBS, 0.1% human holo-transferrin, 1% antibiotic antimycotic, and 1% sodium pyruvate in a humidified incubator with 5% CO_2_ at 37 °C. The cells were sub-cultured at a density of 2 × 10^5^ cells/flask in a 75 cm^2^ flask for 5–6 days.

### 4.12. Preparation of Different Molecular Fragments of VAWEs

The lyophilized powder of SVAE and RVAE was dissolved in sterilized water and then separated by UF concentrator*^®^* (Amicon Ultra-15 Centrifugal Filter Devices, Sigma-Aldrich, St. Louis, MO, USA) by centrifugation at 12,000× *g* for 30 min to obtain the VAWE extraction with different molecular weights greater than 30 kDa, between 3 to 30 kDa, and less than 3 kDa, respectively. The volume concentrated during the centrifugation was made up with sterilized water.

### 4.13. Measurement of CCL-20 Production

Caco-2 cells were seeded at a density of 1.0 × 10^6^ cells/well into 48-well plates overnight. The medium was replaced with DMEM containing VAWE samples at the dosage to be tested with different molecular fragments for 24 h. After the incubation, the supernatants were collected by centrifugation at 1500× *g* for 10 min and quantified by DuoSet ELISA Development Systems (R&D Systems, Minneapolis, MN, USA) according to the manufacturer’s instructions.

### 4.14. Untargeted Metabolomics Analysis of Ultra-High Performance Liquid Chromatography-Mass/Mass (UHPLC-MS/MS) and Gas Chromatography-Mass/Mass (GC-MS/MS)

For the UHPLC-MS/MS analysis, 100 μL of SVAE and RVAE were mixed with the internal standard and placed at −20 °C for 1 h. After centrifugation at 12,000× *g* for 15 min (Heraeus Fresco17, Thermo Fisher Scientific, Waltham, MA, USA), 425 μL of the supernatant was collected and dehydrated by lyophilization. Then, 200 μL of the samples were dissolved in the acetonitrile and water at a 1:1 ratio, and extracted for 30 s. The samples were soaked in an ultrasonic cleaner with water at 4 °C for 15 min of cooling. Finally, the supernatant was collected after centrifugation at 12,000× *g* for 15 min for analysis (75 μL) on a 1290 UHPLC (Agilent, Germany), Triple TOF 6600 Mass (AB Sciex, Framingham, MA, USA), and ACQUITY UPLC BEH Amide column (1.7 μm, 2.1 mm × 100 mm) (Waters, Milford, MA, USA). The parameters of UHPLC-MS/MS are listed in Table S3. For the GC-MS/MS analysis, 50 μL of SVAE and RVAE were mixed with 200 μL of methanol and 5 of μL l-2-chlorophenylalanine for 30 s and placed in the ultrasonic cleaner for 10 min. After centrifugation at 12,000× *g* for 15 min, 180 μL of the supernatant was collected and dehydrated by lyophilization. Then, 80 μL of the samples were dissolved in the pyridine of the methoxide reagent at a 1:1 ratio, and heated for 30 min. Derivatization occurred with 100 μL of *N*, *O*-Bis (trimethylsilyl) trifluoroacetamide (BSTFA) at 70 °C for 90 min. The sample was analyzed on a 7890B GC (Agilent, Waldbronn, Germany), PEGASUS BT Mass (LECO, Germany) with DB-5MS column (30 m × 250 μm × 0.25 μm) (Agilent, Waldbronn, Germany). The parameters of GC-MS/MS are listed in Table S4. Representative samples of SVAE and RVAE (Pooling samples from 3 batches of SVAE or RVAE) were tested. The molecules identified were ranked according to their content, then the top-ranked overlapping components were identified, and the components from the top-ranked computational quotient from RVAE divided by SVAE were further selected for analysis.

### 4.15. Statistical Analysis

For in vitro and in vivo studies, values are given as mean ± standard deviation. All data were analyzed by Student’s t-test and compared to the negative control group (DSS); *p* < 0.05 was considered statistically significant. Nonparametric Mann-Whitney U tests were performed for next-generation sequencing (NGS) data. Correlations were assessed by Spearman’s correlation analysis. All figures were plotted using GraphPad Prism 7.00 software (San Diego, CA, USA).

## 5. Conclusions

In conclusion, both high-dose SVAE and RVAE ameliorate the symptoms of colitis in the DSS-induced mouse model. The underlying mechanism appears to affect the systematic immune response by reducing Th1-related pro-inflammatory cytokines, augmenting barrier function by restoring the tight junction proteins, and improving gut dysbiosis via re-establishing intestinal microflora. The potentially bioactive components of SVAE and RVAE were small molecules (<3 kDa) and included l-carnitine, hypoxanthine, adrenic acid, creatinine, gamma-aminobutyryl-lysine, oleic acid, glycine, poly-γ-glutamic acid, and eicosapentaenoic acid in VAWEs. Further studies are required to verify the anti-colitis effects of these bioactive compounds. Our findings provide new insight into the pharmacological potential of VAWEs for the prevention of colitis.

## Figures and Tables

**Table 1:** The top 20 identified molecules of SVAE and RVAE from UHPLC-MS/MS in positive ion mode.

Rank	SVAE	RVAE
1	Palmitic acid	Palmitic acid
2	l-Carnitine	l-Carnitine
3	Hypoxanthine	Hypoxanthine
4	Creatinine	Creatinine
5	TranexamicAcid; (*S*)-omostachydrine; Homostachydrine; Lentiginosine; Tranexamic acid	TES; *N*-Tris(hydroxymethyl)methyl-2-aminoethanesulfonic acid
6	Acetylcarnitine	2-Methylbutyroylcarnitine
7	2-Methylbutyroylcarnitine	Acetylcarnitine
8	TES; *N*-Tris(hydroxymethyl)methyl-2-aminoethanesulfonic acid	Gamma-Aminobutyryl-lysine
9	Gamma-Aminobutyryl-lysine;	Porson; Gingerenone B; Isogingerenone B; Burseran; (+)-Burseran; (+)-Eudesmin; Pinoresinol dimethyl ether
10	PC(16:0/18:1(9*Z*)); PC(14:0/20:1(11*Z*)); PC(20:0/14:1(9*Z*)); PE(22:1(13*Z*)/15:0); PC(18:1(9*Z*)/16:0); PC(20:1(11*Z*)/14:0); PC(18:0/16:1(9*Z*)); PC(18:1(11*Z*)/16:0); PC(14:1(9*Z*)/20:0); PC(16:0/18:1(11*Z*)); PC(16:1(9*Z*)/18:0); PE(15:0/22:1(13*Z*));	(13*R*,14*R*)-7-Labdene-13,14,15-triol; (13*R*,14*R*)-8-Labdene-13,14,15-triol; (*Z*)-15-Oxo-11-eicosenoic acid
11	Glycerophosphocholine	Thioetheramide-PC
12	Porson; Gingerenone B; Isogingerenone B; Burseran; (+)-Burseran; (+)-Eudesmin; Pinoresinol dimethyl ether	PC(16:0/18:1(9*Z*)); PC(14:0/20:1(11*Z*)); PC(20:0/14:1(9*Z*)); PE(22:1(13*Z*)/15:0); PC(18:1(9*Z*)/16:0); PC(20:1(11*Z*)/14:0); PC(18:0/16:1(9*Z*)); PC(18:1(11*Z*)/16:0); PC(14:1(9*Z*)/20:0); PC(16:0/18:1(11*Z*)); PC(16:1(9*Z*)/18:0); PE(15:0/22:1(13*Z*));
13	Erucamide	Glycerophosphocholine
14	Thioetheramide-PC	3-(2-Hydroxyethyl)indole
15	N1-(3-Aminopropyl)agmatine; N1-Aminopropylagmatine	4-*O*-Methylmelleolide; Clausarinol; Eplerenone; Armillarin; Armillaripin; Magnoshinin; Eplerenone; Estra-1,3,5(10)-triene-3,6alpha,17beta-triol triacetate;
16	4-*O*-Methylmelleolide; Clausarinol; Eplerenone; Armillarin; Armillaripin; Magnoshinin; Eplerenone; Estra-1,3,5(10)-triene-3,6alpha,17beta-triol triacetate; Estra-1,3,5(10)-triene-3,6beta,17beta-triol triacetate	l-Leucine
17	*O*-Phosphotyrosine; Phosphotyrosine; Phosphonotyrosine	Isobutylpropylamine
18	Isobutylpropylamine	1-Methylhistidine
19	(3-Carboxypropyl)trimethylammonium cation	Threoninyl-Lysine; Lysyl-Threonine
20	Threoninyl-Lysine; Lysyl-Threonine	Cytidine

**Table 2:** The top 20 identified molecules of SVAE and RVAE from UHPLC-MS/MS in negative ion mode.

Rank	SVAE	RVAE
1	Oleic acid	Oleic acid
2	Palmitic acid	*cis*-9-Palmitoleic acid
3	Arachidonic Acid (peroxide free)	Palmitic acid
4	*cis*-9-Palmitoleic acid	Arachidonic Acid (peroxide free)
5	Suberenone; Graveolone; Eriobofuran; (2*E*,11*Z*)-Wyerone acid; 9,10-Dihydro-2,3,5,7-Phenanthrenetetrol; 3,3′,4′5-Tetrahydroxystilbene; (*R*)-Apiumetin; Piceatannol; 3,3′,4′5-Tetrahydroxystilbene; Wyerone acid; Eriobofuran; 2,4-Dimethoxydibenzofuran-3-ol; Fulvoplumierin; Oxyresveratrol; Methylstyrylpyron; 2,2′-Dihydroxy-4-methoxybenzophenone; Dioxybenzone	Uracil
6	Isoplumbagin; 1-Hydroxy-2-phthoate; 1-Hydroxy-2-phthoic acid; 1-phthol-2-carboxylic acid; Plumbagin; Ramentaceone; 7-Methyljuglone; 3-Hydroxy-2-phthoate	Hypoxanthine
7	Hypoxanthine	Myristoleic acid
8	Pyridine N-oxide glucuronide; 16-Hydroxypalmitate; 16-Hydroxypalmitic acid	Isoplumbagin; 1-Hydroxy-2-phthoate; 1-Hydroxy-2-phthoic acid; 1-phthol-2-carboxylic acid; Plumbagin; Ramentaceone; 7-Methyljuglone; 3-Hydroxy-2-phthoate
9	Isoferulic acid 3-sulfate; Ferulic acid 4-sulfate	Suberenone; Graveolone; Eriobofuran; (2*E*,11*Z*)-Wyerone acid; 9,10-Dihydro-2,3,5,7-Phenanthrenetetrol; 3,3′,4′5-Tetrahydroxystilbene; (*R*)-Apiumetin;Piceatannol; 3,3′,4′5-Tetrahydroxystilbene; Wyerone acid;Eriobofuran; 2,4-Dimethoxydibenzofuran-3-ol; Fulvoplumierin; Oxyresveratrol; Methylstyrylpyron; 2,2′-Dihydroxy-4-methoxybenzophenone; Dioxybenzone
10	Dihomo-gamma-Linolenic Acid	Isoferulic acid 3-sulfate; Ferulic acid 4-sulfate
11	dl-lactate	Atenolol; Practolol; Tributyl phosphate; TBP
12	7Z, 10Z, 13Z, 16Z, 19Z-Docosapentaenoic acid	Dihomo-gamma-Linolenic Acid
13	Pentadecanoic Acid	dl-lactate
14	Uracil	9*R*,10*S*-EpOME
15	Pristimerin	7*Z*, 10*Z*, 13*Z*, 16*Z*, 19*Z*-Docosapentaenoic acid
16	3alpha-Hydroxy-3,5-dihydromocolin L acid	3alpha-Hydroxy-3,5-dihydromocolin L acid
17	Atenolol; Practolol; Tributyl phosphate; TBP	12(*R*)-HETE
18	Adrenic Acid	2-Oxoadipic acid
19	12(*R*)-HETE	Pentadecanoic Acid
20	Bromobenzene	Adrenic Acid

**Table 3:** The top 20 identified molecules of SVAE and RVAE from GC-MS/MS.

Rank	SVAE	RVAE
1	lactic acid	lactic acid
2	alanine	alanine
3	palmitic acid	urea
4	glycine	methylamine
5	methylamine	galactose
6	urea	glycine
7	proline	palmitic acid
8	stearic acid	proline
9	galactose	isoleucine
10	isoleucine	oxoproline
11	oxoproline	valine
12	valine	stearic acid
13	glutamic acid	glutamic acid
14	isoleucine	glycine 1
15	glycine 1	isoleucine
16	hypoxanthine	uracil
17	serine	3-hydroxybutyric acid
18	glycolic acid	hypoxanthine
19	aminomalonate	glycolic acid
20	glyceric acid	glyceric acid

## Data Availability

Not applicable.

## Supplementary Materials

The following supporting information can be downloaded at: https://www.mdpi.com/article/10.3390/biomedicines11071913/s1, Table S1: The identified molecules of top-ranked computational fold changes from RVAE divided by SVAE; Table S2: The identified molecules of top-ranked computational fold changes from SVAE divided by RVAE; Table S3: The condition of mobile phase of ultra-high performance liquid chromatography; Table S4: The parameter of ultrahigh performance gas chromatography; Figure S1: Effects of VAWEs on (A) the record of body weight, (B) the food intake and (C) the feces occult bleeding scores evaluation; Figure S2: Effects of different VAWEs on the microbiome analysis in DSS-induced colitis mice model of (A) chao1 richness estimator and Shannon’s diversity index of alpha-diversity and (B) PLS-DA plots of the individual mice cluster of beta-diversity.
