# Supplementary material for: Multiomics Strategy Reveals the Mechanism of Action and Ameliorating Effect of Deer Velvet Antler Water Extracts on DSS-Induced Colitis
Source: Biomedicines. 2023 Jul 6;11(7):1913. doi: 10.3390/biomedicines11071913 (PMC10377209; doi:10.3390/biomedicines11071913)
Supplement: Supplementary file 1 [file biomedicines-11-01913-s001.zip › Supplementary_Table_and_fig_R.pdf]

Supplementary Information

Running title:

Effect and mechanism of deer velvet antler water extracts on inflammatory bowel disease

Multiomics strategy reveals the mechanism of action and ameliorating effect of deer velvet antler water extracts on DSS-induced Colitis

Ying-Kai Hung [1, #], Shang-Tse Ho [2, #], Ching-Yun Kuo [3, *], and Ming-Ju Chen [1, *]

[1] Department of Animal Science and Technology, National Taiwan University, Taipei 106, Taiwan

[2] Department of Wood Based Materials and Design, National Chiayi University, Chiayi 600, Taiwan

[3] Taiwan Livestock Research Institute, Council of Agriculture, Tainan 712, Taiwan

---

*Corresponding author: Ming-Ju Chen and Ching-Yun Kuo

[#]These authors contributed equally to the work

Mailing address: Department of Animal Science and Technology, National Taiwan University, No. 50, Lane 155, Sec. 3. Keelung Rd., Taipei 106, Taiwan

Phone number: 886-2-33664173

E-mail: MJC: cmj@ntu.edu.tw; CYK: kuochingyun@gmail.com

Contents

Table S1. The identified molecules of top-ranked computational fold changes from RVAE divided by SVAE 3

Table S2. The identified molecules of top-ranked computational fold changes from SVAE divided by RVAE 4

Table S3. The condition of mobile phase of ultra-high performance liquid chromatography 5

Table S4. The parameter of ultrahigh performance gas chromatography 6

Figure S1. Effects of VAWEs on (A) the record of body weight, (B) the food intake and (C) the feces occult bleeding scores evaluation. 7

Figure S2. Effects of different VAWEs on the microbiome analysis in DSS-induced colitis mice model of (A) chao1 richness estimator and Shannon's diversity index of alpha-diversity and (B) PLS-DA plots of the individual mice cluster of beta-diversity. 9

Table S1. The identified molecules of top-ranked computational fold changes from RVAE divided by SVAE

Rank	Identified components	Fold Changes
1	Campesterol 6'-hexadecanoylglucoside	
2	CE(20:5(5*Z*,8*Z*,11*Z*,14*Z*,17*Z*)	
3	*N*-Octanoyl-L-homoserine lactone;C8-HSL; *N*-[(3S)-Tetrahydro-2-oxo-3-furanyl]octamide	328.5243
4	L-Leucine	313.6711
5	*N*-Phosphohypotaurocyamine; N(omega)-Phosphohypotaurocyamine	134.1230
6	N6-Carbamoyl-L-threonyladenosine; Cyflufemid	131.7877
7	3',5'-Diiodo-L-thyronine-beta-D-glucuronoside	130.3344
8	8-Hydroxy-*R*-acenocoumarol; 6-Hydroxy-*R*-acenocoumarol; 7-Hydroxy-*R*-acenocoumarol; *S*-(2-Chloroethyl)glutathione	129.9808
9	Zanthobisquinolone; gilactone C; Vernolide; Gibberellin A8-catabolite	117.4303
10	Ethyl 3,4,5-trimethoxybenzoate; 3-Carboxy-4-methyl-5-propyl-2-furanpropionic acid; 3-(3,4,5-Trimethoxyphenyl)propanoic acid; Isopropyl 3-(3,4-dihydroxyphenyl)-2-hydroxypropanoate; 2-Hydroxyamino-1-methyl-6-phenylimidazo[4,5-b]pyridine; 2-Hydroxyamino-PhIP	113.0509
11	Haemocorin	100.7021
12	8-Hydroxyclomipramine; Clomipramine N-oxide; Hydroxyclomipramine; 2-Hydroxyclomipramine; Podolide; Gibberellin A51-catabolite; Gibberellin A7; Gibberellin A5; 5-Deoxystrigol	98.9067
13	Eriocitrin; Isorubrofusarin 10-gentiobioside; Rubrofusarin 6-gentiobioside; (2*R*)-6,8-Diglucopyranosyl-4',5,7-trihydroxyflavanone; Cassiaside C; Pinobanksin 5-[galactosyl-(1->4)-glucoside]; Isobutrin; Butrin; Butin 7,3'-di-*O*-glucoside; Eriocitrin; Eriodictyol 7-*O*-rutinoside; Neoeriocitrin; Eriodictyol 7-*O*-neohesperidoside	92.6039
14	1-Palmitoyl-2-(5-keto-6-octendioyl)-sn-glycero-3-phosphatidylcholine	86.1986
15	D-Dethiobiotin	78.9208
16	4-{[4-(Diethylamino)phenyl](2,4-disulfophenyl)methylene}-*N*,*N*-diethyl-2,5-cyclohexadien-1-iminium	70.5839
17	Cyclamate; Sodium cyclamate	68.2191
18	Erucin;1-Thiocyanato-4-(methylthio)butane;*N*-Formyl-L-aspartate	67.2622
19	Eicosapentaenoic Acid	65.2990
20	2-Methyl-1,4-naphthalenediol bis(dihydrogen phosphate); 9-Fluoro-16alpha-hydroxyandrost-4-ene-3,11,17-trione	64.6659
21	Maclurin 3-C-(2"-galloyl-6"-p-hydroxybenzoyl-glucoside); Maclurin 3-C-(2"-p-hydroxybenzoyl-6"-galloyl-glucoside)	52.9826

Table S2. The identified molecules of top-ranked computational fold changes from SVAE divided by RVAE

Rank	Identified components	Fold Changes
1	Cholesteryl sulfate	235.5627
2	2'-O-Methyladenosine; 1-Methyladenosine; 3'-O-Methyladenosine; N6-Methyladenosine; 2,3,3-Triphenylacrylonitrile; Triphenylcyanoethylene	112.3688
3	Plakortic acid	104.3961
4	PC(22:2(13Z,16Z)/16:0); PC(24:1(15Z)/14:1(9Z)); PC(14:1(9Z)/24:1(15Z)); PC(20:1(11Z)/18:1(11Z)); PC(18:1(11Z)/20:1(11Z)); PC(16:0/22:2(13Z,16Z)); PC(18:2(9Z,12Z)/20:0); PC(20:1(11Z)/18:1(9Z)); PC(22:1(13Z)/16:1(9Z)); PC(18:0/20:2(11Z,14Z)); PC(20:2(11Z,14Z)/18:0); PC(16:1(9Z)/22:1(13Z)); PC(18:1(9Z)/20:1(11Z)); PC(20:0/18:2(9Z,12Z))	64.1723
5	5,2'-O-dimethylcytidine	58.4683
6	Nicotinuric acid	51.6283
7	Selenomethyl selenocysteine; Se-Methylselenocysteine; Selenohomocysteine; Se-Methyl-L-selenocysteine; Selenohomocysteine	41.7061
8	1,2-Diamino-4-nitrobenzene; 1,4-Diamino-2-nitrobenzene	40.0792
9	L-Carnitine	39.4910
10	Linalyl anthranilate; alpha-Terpinyl anthranilate; (2R,3R)-3-Methylglutamyl-5-semialdehyde-N6-lysine	31.8243
11	3''-Hydroxy-geranylhydroquinone	31.7275
12	Lenalidomide; Litine; N-(gamma-L-Glutamyl)amino-D-proline	31.2021
13	Dichloromaleimide; Oxalosuccite; Oxalosuccinic acid	30.7499
14	N-Desmethyl-p-O-sulfate rosiglitazone; N-Desmethyl-o-O-sulfate rosiglitazone; Glucolimnthin; Glucoaubrietin; 4-Methoxybenzyl glucosinolate; Glucobarbarin;2(R)-Hydroxy-2-phenylethyl glucosinolate	26.1064
15	Isopentenyladenosine	23.9937
16	Phlorisobutyrophenone 2-glucoside; Tarennoside; 10-Deoxygeniposidic acid; Sweroside	22.6067
17	Ala-Pro	20.9027
18	Ethalfluralin	20.1360
19	dUMP; Nimustine hydrochloride; ACNU	20.0223

Table S3. The condition of mobile phase of ultra-high performance liquid chromatography

minutes	rate (μL/min)	Solvent A (25mM ammonium acetate with 25mM ammonium mixture)	Solvent B (Acetonitrile)
0	500	5	95
0.5	500	5	95
7	500	35	65
8	500	60	40
9	500	60	40
9.1	500	5	95
12	500	5	95

Table S4. The parameter of ultrahigh performance gas chromatography

Items	Parameters
sample volume	1 μL
front inlet mode	Split Mode
front inlet septum purge flow	3 mL/min
carrier gas	Helium
column	DB-5MS (30 m × 250 μm × 0.25 μm)
column flow	1 mL/min
oven temperature ramp	50˚C for 0.5 min, raised to 320℃ in 15℃/min
front injection temperature	280˚C
transfer line temperature	320˚C
ion source temperature	230˚C
electron energy	-70 eV
Mass range	m/z: 75-650
acquisition rate	10 spectra/sec
solvent delay	3.833 min

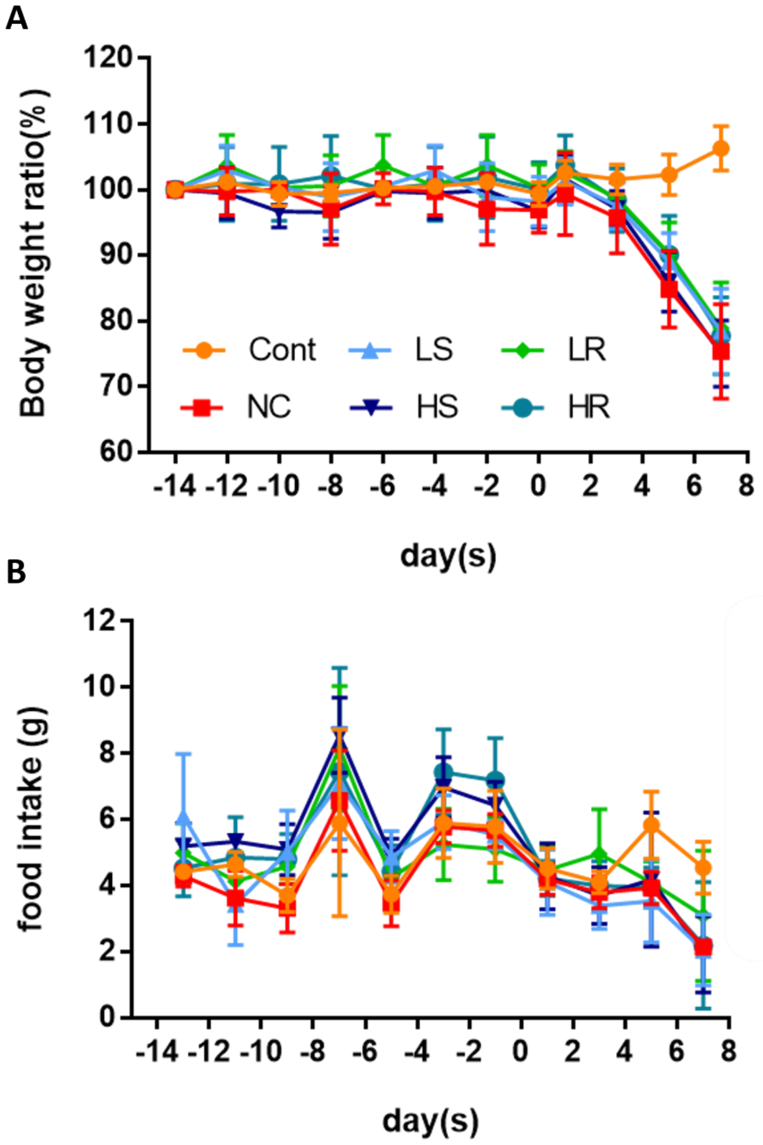

Figure S1. Effects of VAWEs on (A) the record of body weight, (B) the food intake and (C) the feces occult bleeding scores evaluation.

A

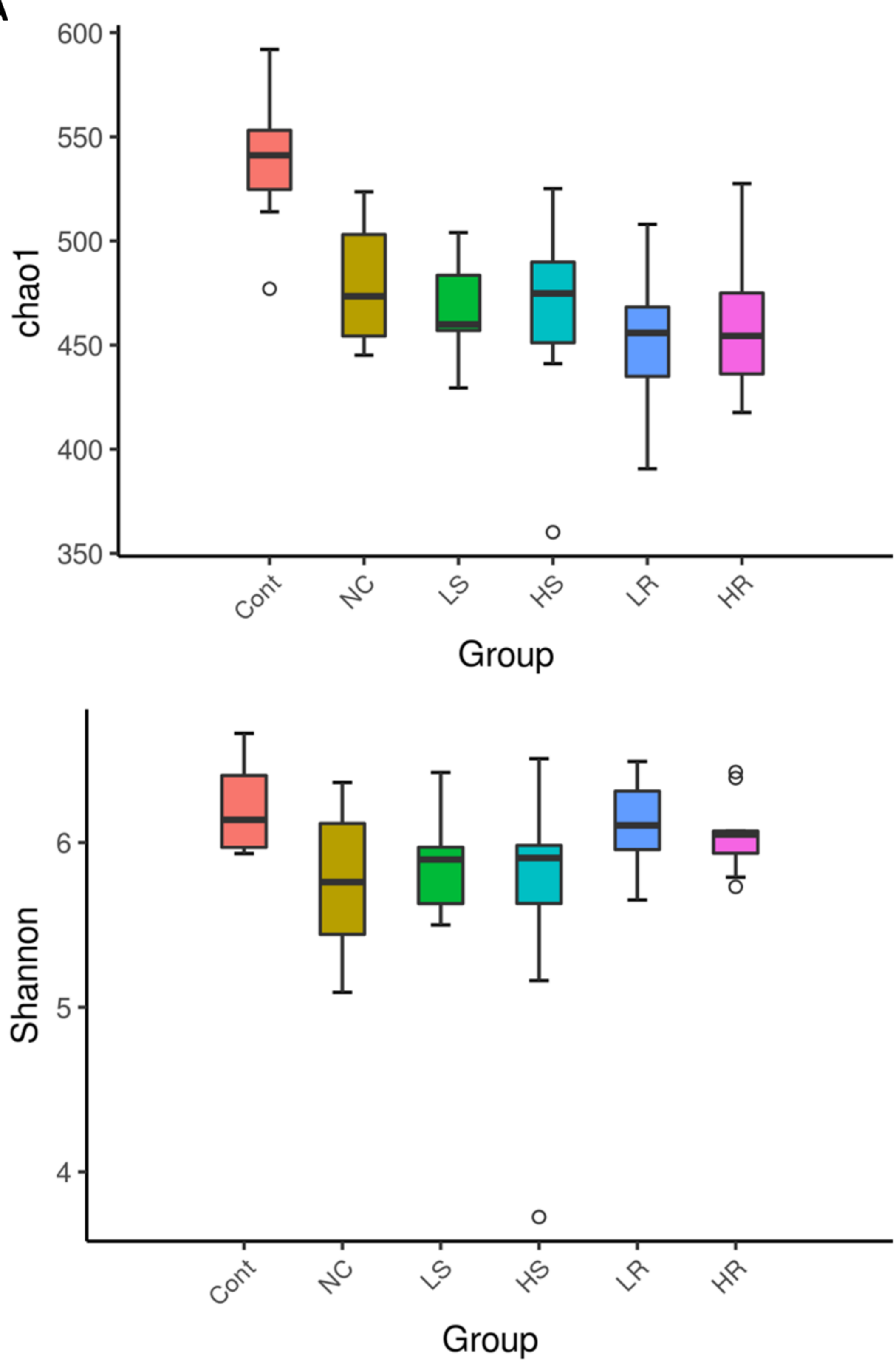
600
550
500
450
400
350
chao1
Cont
NC
LS
HS
LR
HR
Group
6
5
4
Shannon
Cont
NC
LS
HS
LR
HR
Group

B

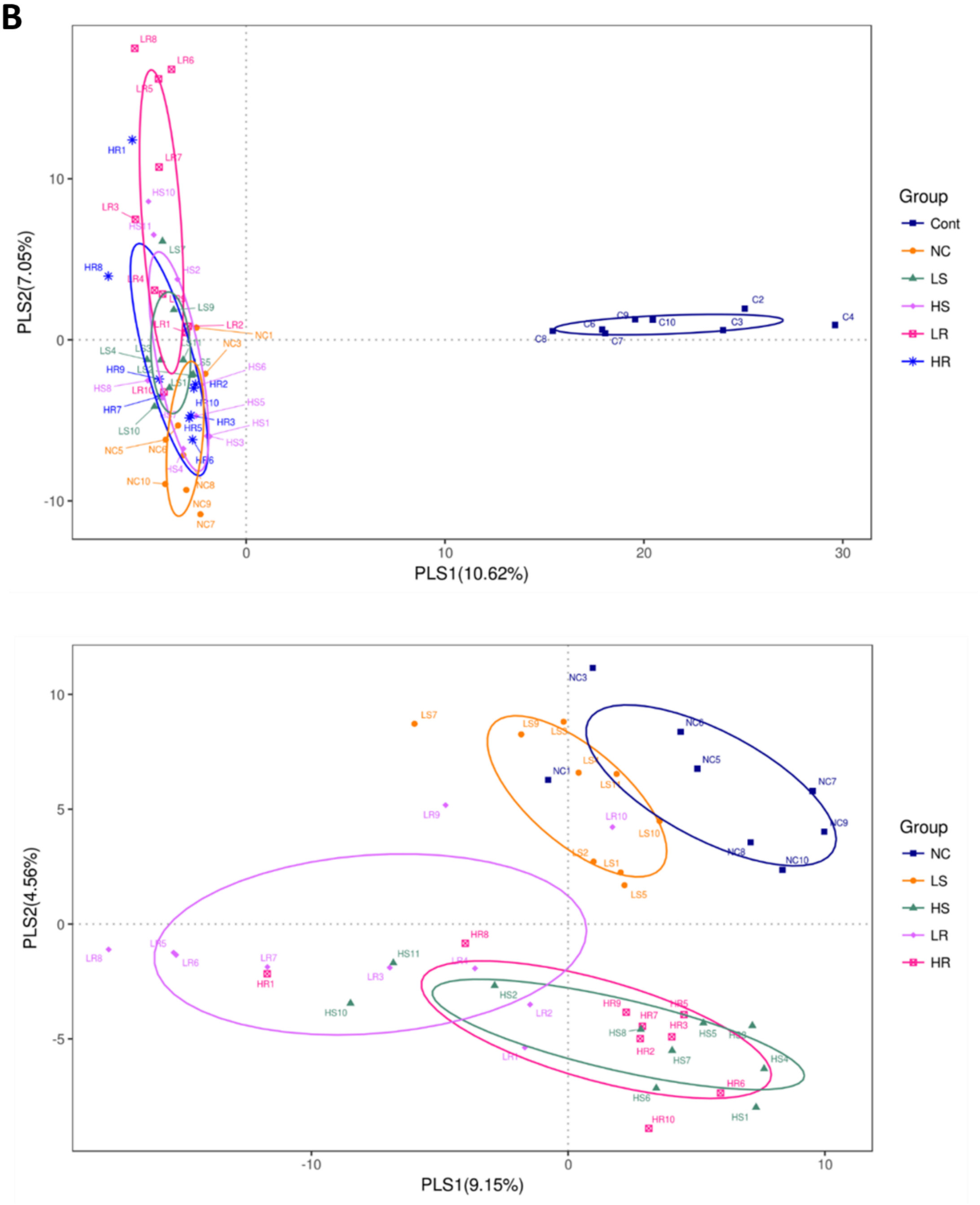

Figure S2. Effects of different VAWEs on the microbiome analysis in DSS-induced colitis mice model of (A) chao1 richness estimator and Shannon's diversity index of alpha-diversity and (B) PLS-DA plots of the individual mice cluster of beta-diversity.
