# Supplementary material for: Multiomics Strategy Reveals the Mechanism of Action and Ameliorating Effect of Deer Velvet Antler Water Extracts on DSS-Induced Colitis
Source: Biomedicines. 2023 Jul 6;11(7):1913. doi: 10.3390/biomedicines11071913 (PMC10377209; doi:10.3390/biomedicines11071913)

Figure 3(C). Western blot analysis of occludin (59kDa)

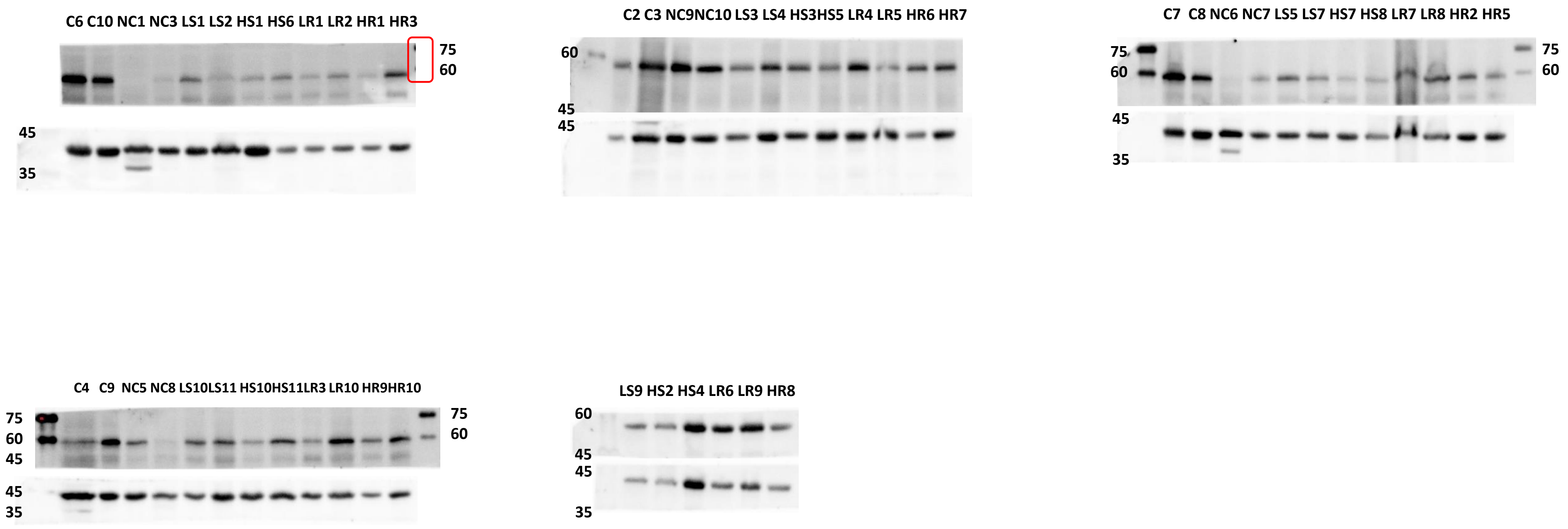

Figure 3(C). Western blot analysis of claudin-1 (23kDa)

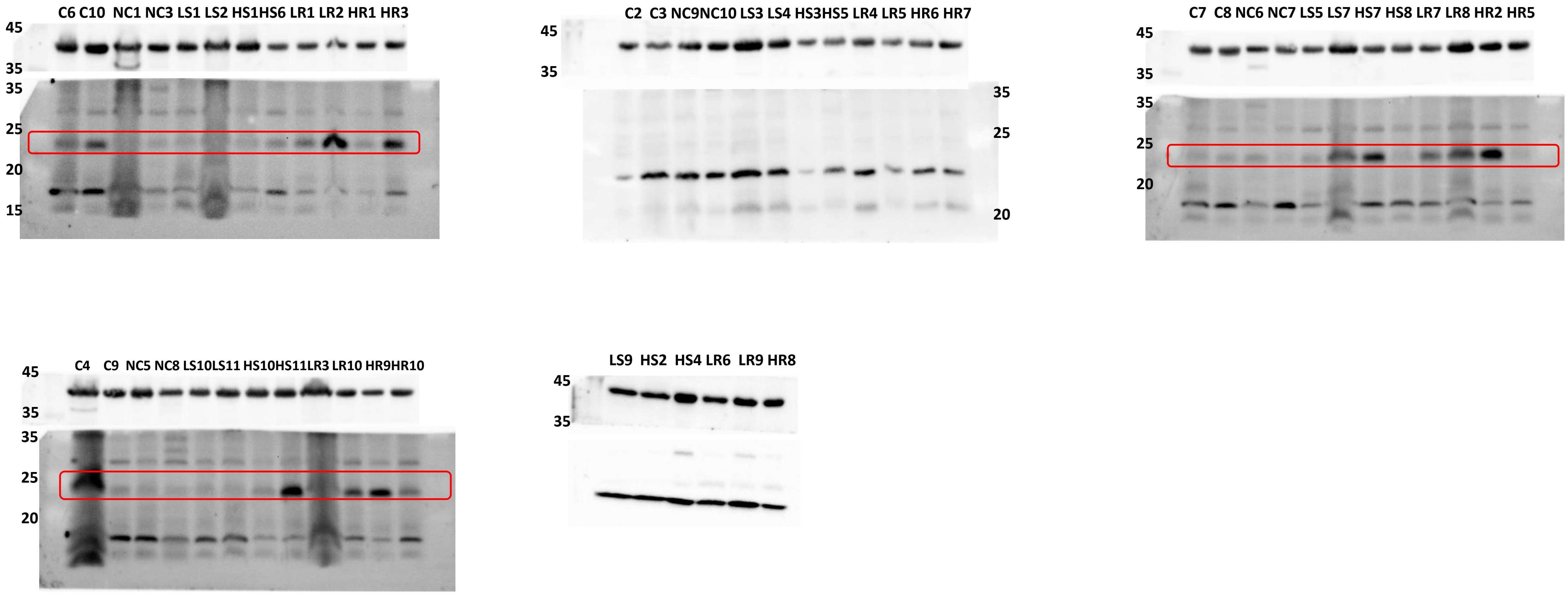

Figure 3(C). Western blot analysis of claudin-2 (25kDa)

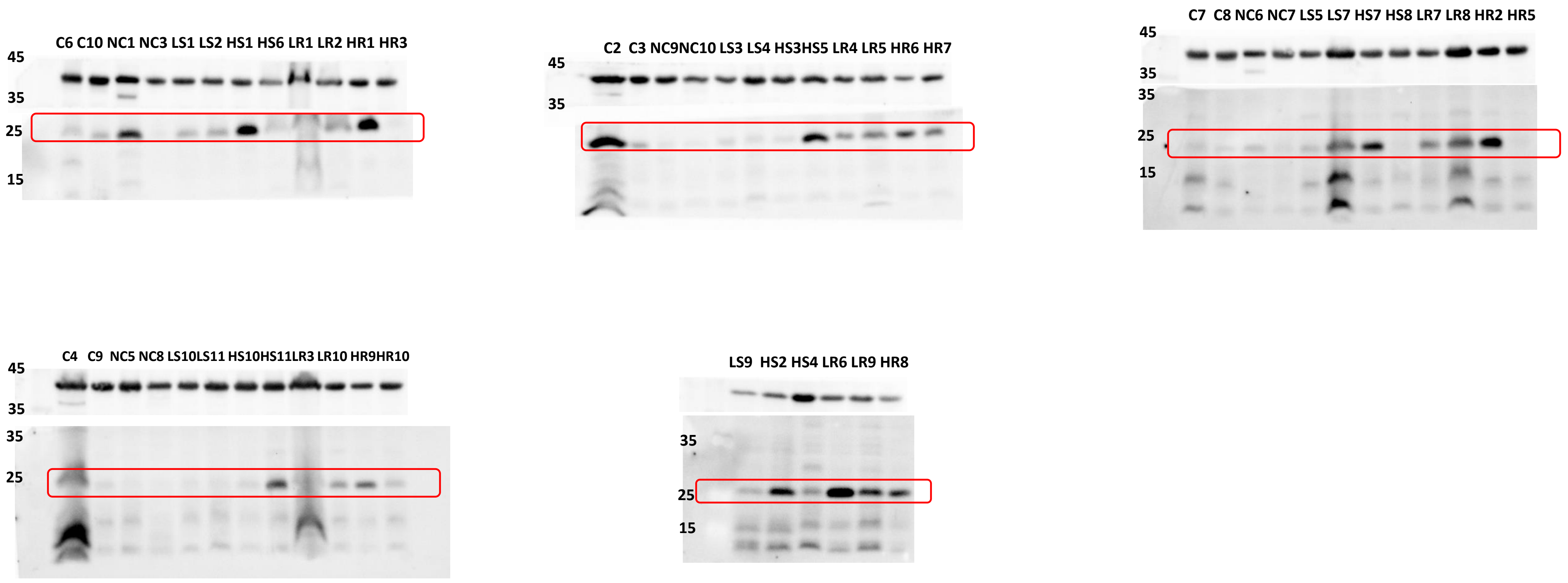

Figure 3(C). Western blot analysis of claudin-4 (22kDa)

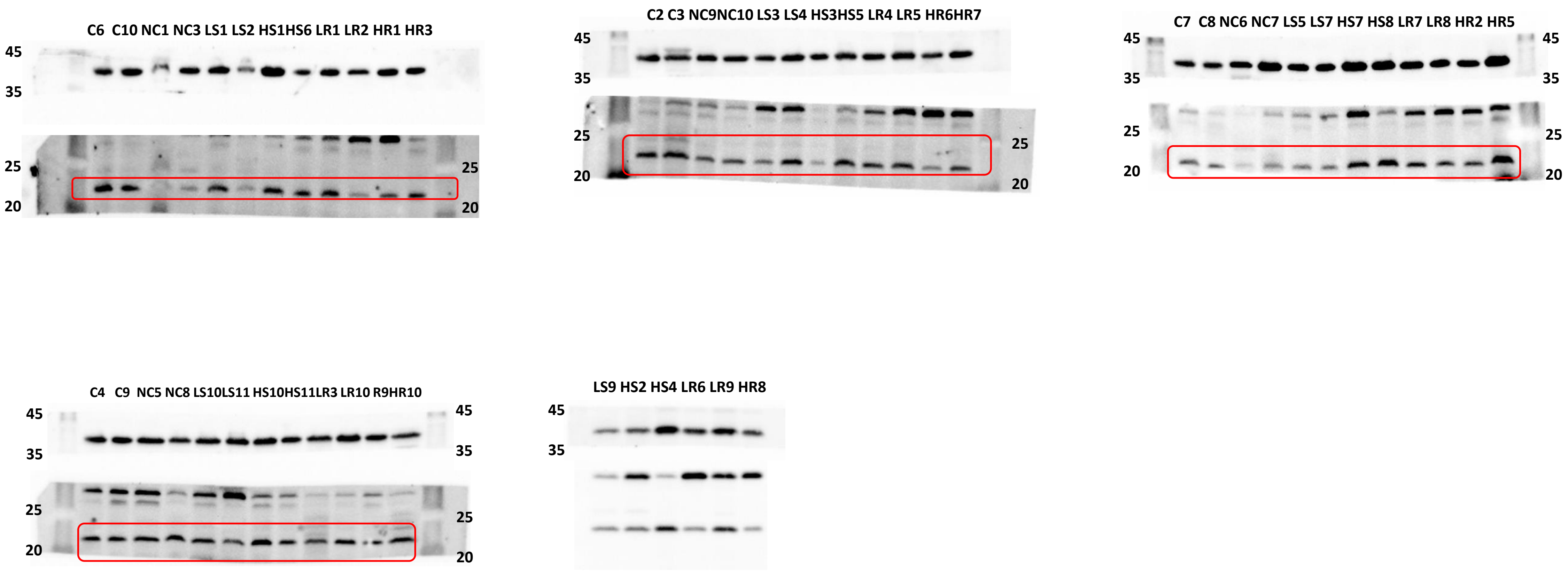

Figure 3(C). Western blot analysis of ZO-1 (187kDa)

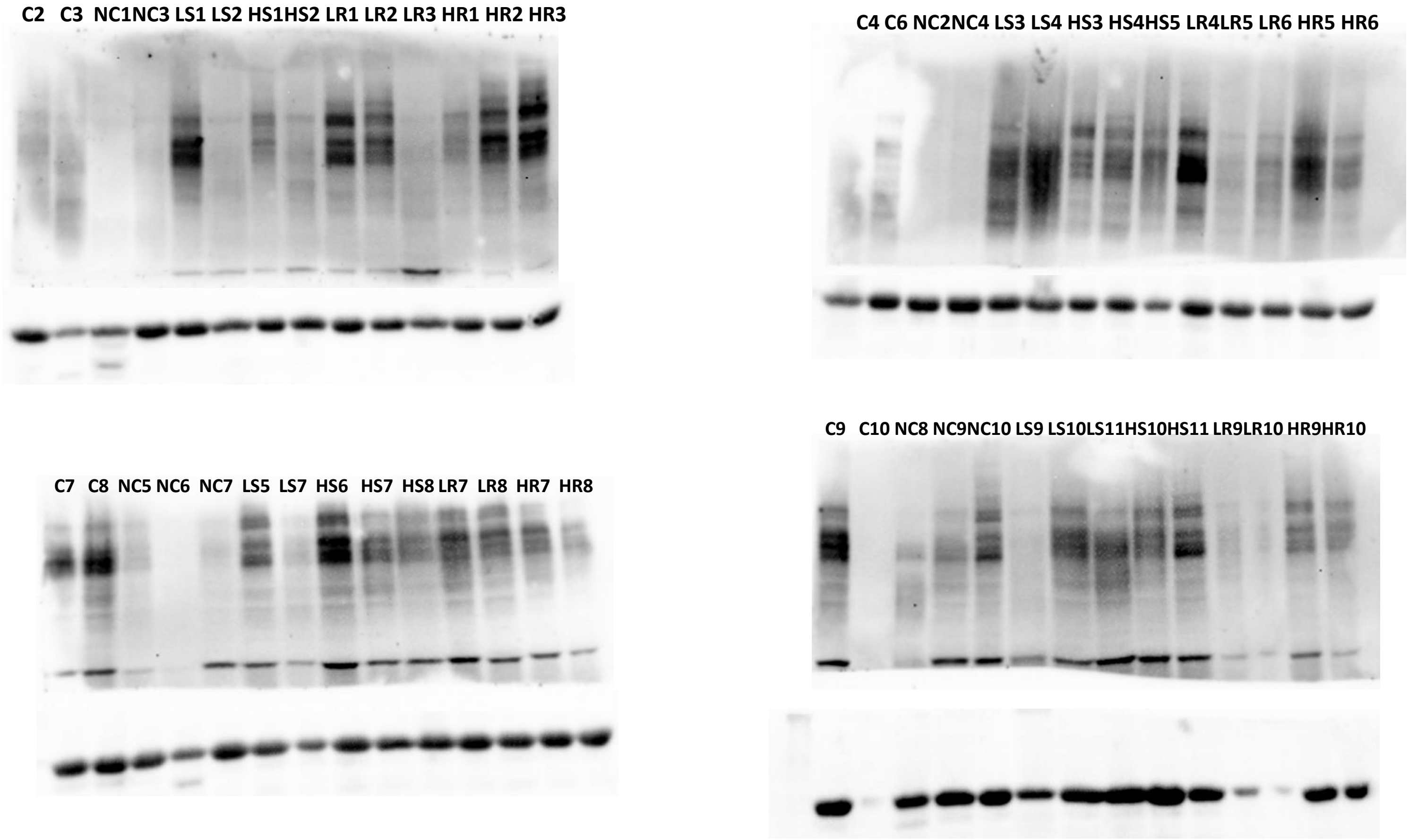

Figure 4(B). Western blot analysis of colonic HIF-1α (93kDa)

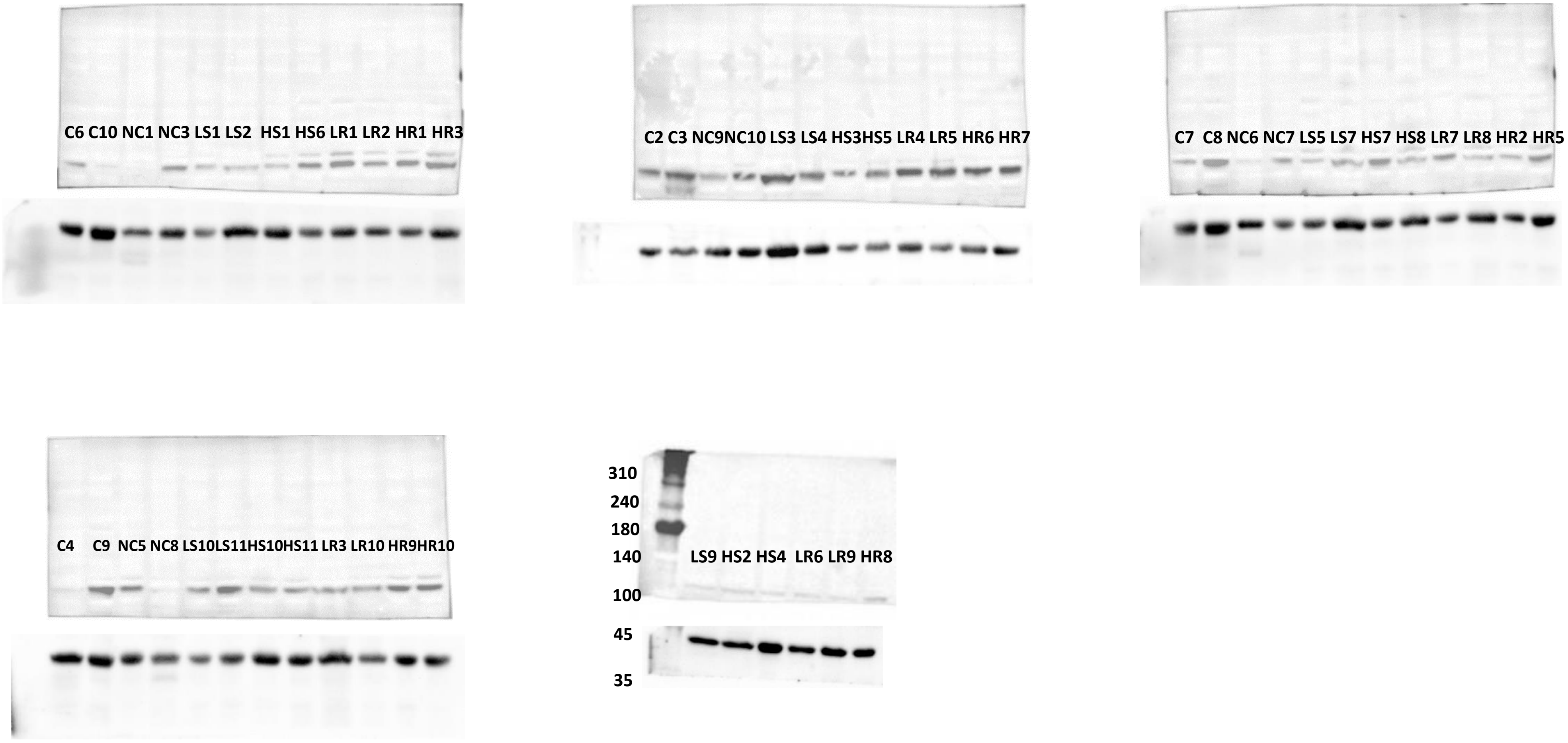

Figure 4(B). Western blot analysis of colonic HIF-1α (93kDa)

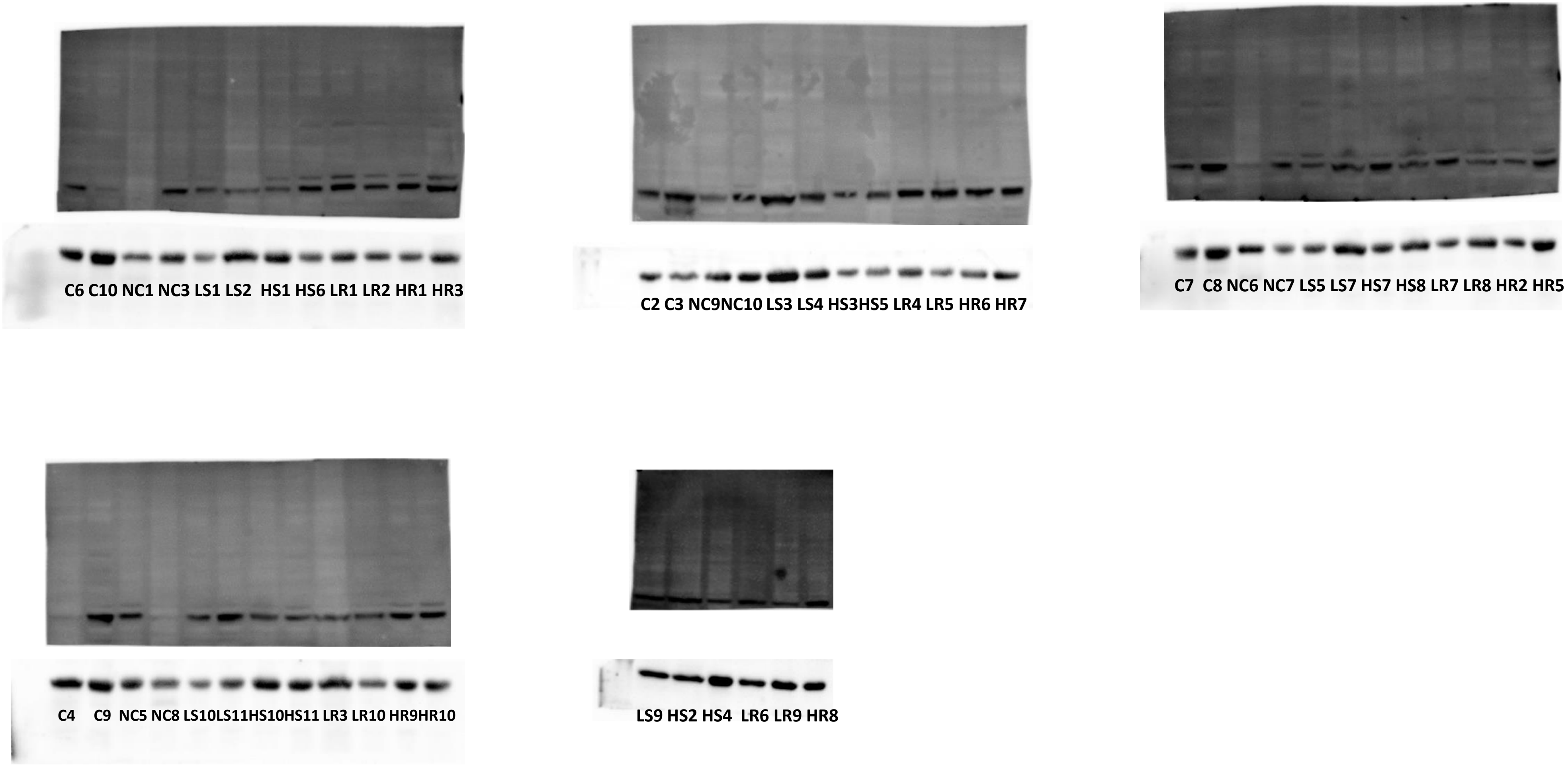

Supplement: Supplementary file 1 [file biomedicines-11-01913-s001.zip › western-blotting-raw-data.pdf]
